# Stromal fibroblasts support dendritic cells to maintain IL-23/Th17 responses after exposure to ionizing radiation

**DOI:** 10.1189/jlb.3A1015-474R

**Published:** 2016-04-05

**Authors:** Anna Malecka, Qunwei Wang, Sabaria Shah, Ruhcha V. Sutavani, Ian Spendlove, Judith M. Ramage, Julie Greensmith, Hester A. Franks, Michael J. Gough, Anja Saalbach, Poulam M. Patel, Andrew M. Jackson

**Affiliations:** * Host-Tumour Interactions Group, University of Nottingham, Nottingham, United Kingdom;; † Cancer Immunotherapy Group, Division of Cancer and Stem Cells, University of Nottingham, Nottingham, United Kingdom;; ‡ Cell Signalling and Immunology, University of Dundee, Scotland, United Kingdom;; § Intelligent Modelling and Analysis Research Group, University of Nottingham, Nottingham, United Kingdom; ¶ Earle A. Chiles Research Institute, Robert W. Franz Cancer Center, Providence Portland Medical Center, Portland, Oregon, USA;; ‖ Klinik fur Dermatologie, University of Leipzig, Germany; and

**Keywords:** cytokine, immunity, cancer

## Abstract

Dendritic cell function is modulated by stromal cells, including fibroblasts. Although poorly understood, the signals delivered through this crosstalk substantially alter dendritic cell biology. This is well illustrated with release of TNF-α/IL-1β from activated dendritic cells, promoting PGE_2_ secretion from stromal fibroblasts. This instructs dendritic cells to up-regulate IL-23, a key Th17-polarizing cytokine. We previously showed that ionizing radiation inhibited IL-23 production by human dendritic cells in vitro. In the present study, we investigated the hypothesis that dendritic cell-fibroblast crosstalk overcomes the suppressive effect of ionizing radiation to support appropriately polarized Th17 responses. Radiation (1–6 Gy) markedly suppressed IL-23 secretion by activated dendritic cells (*P* < 0.0001) without adversely impacting their viability and consequently, inhibited the generation of Th17 responses. Cytokine suppression by ionizing radiation was selective, as there was no effect on IL-1β, -6, -10, and -27 or TNF-α and only a modest (11%) decrease in IL-12p70 secretion. Coculture with fibroblasts augmented IL-23 secretion by irradiated dendritic cells and increased Th17 responses. Importantly, in contrast to dendritic cells, irradiated fibroblasts maintained their capacity to respond to TNF-α/IL-1β and produce PGE_2_, thus providing the key intermediary signals for successful dendritic cell-fibroblasts crosstalk. In summary, stromal fibroblasts support Th17-polarizing cytokine production by dendritic cells that would otherwise be suppressed in an irradiated microenvironment. This has potential ramifications for understanding the immune response to local radiotherapy. These findings underscore the need to account for the impact of microenvironmental factors, including stromal cells, in understanding the control of immunity.

## Abstract (precis)

Cross talk between DCs and FBs in understanding the effects of IR in DC function.

## Introduction

Crosstalk between DCs and stromal FBs has a marked impact on DC function with regards to their ability to mature, migrate, and trigger appropriate adaptive responses [1–3]. Whereas the importance of crosstalk between FBs and the immune system for the onset and maintenance of cancer and autoimmunity was recently recognized, the exact mechanisms governing these interactions and the extent to which stromal cells affect the outcome of therapeutic interventions, such as RT, remain poorly understood [4–7].

FBs play important roles in the pathology of a wide range of diseases. Tumor-associated FBs promote tolerogenic DCs in hepatic, breast, and ovarian cancers, whereas in pancreatic cancer, they skew DCs to promote Th2 responses [8–11]. Pulmonary FBs are involved in the pathology of chronic obstructive pulmonary disease and asthma by secreting CCL2 and CCL20 to attract DCs to airways and thus, maintain chronic inflammation [12]. Likewise, synovial FBs are implicated in the perpetuation of rheumatoid arthritis through recruitment and activation of leukocytes, including T cells, macrophages, and DCs [7, 13]. Recently, we established that FBs modulate IL-23 secretion by DCs to promote Th17 responses, and this process was implicated in the maintenance and progression of psoriatic lesions [3]. This model proposes that dermal FBs respond to TNF-α and IL-1β, secreted from activated DCs, by producing PGE_2_. FB-derived PGE_2_ acts in a juxtacrine manner to amplify IL-23 release from DCs, thus supporting the generation of Th17 responses. The IL-23/Th17 axis is important for the development and maintenance of autoimmune disorders, including rheumatoid arthritis, psoriasis, and colitis [14–17]. IL-23 promotes tumor growth directly and indirectly through Th17 responses that drive proliferation, invasion, metastasis, and angiogenesis [18–23]. Furthermore, it is implicated in the development of idiopathic and radiation-induced fibrosis [24, 25].

RT is a major tool for the treatment and palliation of tumors, including squamous cell carcinoma of the skin, breast, and primary brain tumors and brain metastases [26–28]. Current protocols use fractionated RT, typically comprising relatively low doses (1–6 Gy) of IR administered over a period of weeks [26, 29] This allows tumor cells to be targeted with sufficient cumulative dose to deliver therapeutic benefit, while restricting side-effects to a tolerable minimum. However, during RT, immune and stromal cells residing in and adjacent to the tumor niche are also affected by IR through direct and bystander effects. Irradiated tumor cells up-regulate MHC class I expression and secrete chemokines and cytokines, such as CXCL16, TNF-α, IL-1β, and IL-6, which in turn, stimulate infiltration and activation of DCs [30]. DCs are relatively resistant to radiation-induced apoptosis, maintaining viability at doses up to 30 Gy [31]. Their radio-resistance is a result of constitutively expressed DNA repair systems, including ATM kinase and DNA-dependent protein kinase [32]. However, despite this, direct exposure of DCs to IR suppresses their function. Irradiation of DCs down-regulates the production of the T cell-polarizing cytokine IL-12 without affecting IL-10, thus changing the ratio between pro- and anti-inflammatory stimuli and shifting the balance from T cell activation to tolerance [31]. Furthermore, we recently described that IR inhibits secretion of IL-23, another T cell-polarizing cytokine, by DCs [33]. Taken together, this suggests that in the setting of local RT, the cytokine “signal 3” from activated DCs is substantially modified, thus altering the nature of subsequent T cell responses.

Therefore, we investigated the hypothesis that local FBs support IL-23 release by irradiated DCs and thereby, maintain their ability to generate Th17 responses. Doses of radiation consistent with those used for fractionated RT selectively inhibited IL-23 and to a lesser extent, IL-12 by DCs, without affecting IL-10, IL-6, IL-27, TNF-α, or IL-1β secretion. Interestingly, IR did not affect the capacity of FBs to amplify IL-23 secretion by DCs. The coculture of irradiated FBs with irradiated DCs up-regulated IL-23 secretion and increased Th17 responses. We examined the factors by which FBs support the function of DCs, despite the presence of IR, by using a DC-FB coculture system in the presence of IR. The enhancing effect of FB was mimicked by addition of PGE_2_ or forskolin to irradiated DCs and was abrogated by treating FBs with the COX2 inhibitor indomethacin. This effect occurred despite activation of the ATM pathway in irradiated DCs, which we previously established was involved in IL-23 down-regulation [33]. These findings indicate that even after exposure to IR, activated FBs act through secretion of PGE_2_ to activate the cAMP pathway in irradiated DCs, leading to increased IL-23 secretion, independent of ATM kinase. Our findings not only establish the significance of FBs in regulating DC responses but also highlight the complex interplay between DCs and their microenvironment and illustrate the importance of developing more comprehensive cell biology models for understanding immunity.

## MATERIALS AND METHODS

### Reagents

All reagents were endotoxin free. rhGM-CSF and rhTNF-α were from PeproTech (Rocky Hill, NJ, USA); IL-4 and IFN-γ were from R&D Systems Europe (Oxford, United Kingdom); Ultrapure TLR4 agonist (*Salmonella Minnesota* LPS) was from InvivoGen (San Diego, CA, USA); rhIL-1β and rhIL-6 were from ImmunoTools (Friesoythe, Germany); IL-23 was from eBioscience (San Diego, CA, USA); and PGE_2_, indomethacin, and forskolin were from Sigma-Aldrich (Dorset, United Kingdom). Mouse anti-human CD4-PE was from BD Biosciences (Oxford, United Kingdom); mouse anti-human CD4-PE-Cy7, mouse anti-human CD45RA-FITC, mouse anti-human CD14-PE-Cy5.5, and matching isotype controls were from eBioscience. For CD4 activation, mouse anti-human CD28 was obtained from BD Biosciences, IL-2 from R&D Systems Europe, and CD3 (OKT3) was produced in-house. The annexin V/PI staining kit was obtained from BD Biosciences.

### Generation of MoDCs

Mo-DCs were generated as described previously [34]. In brief, fresh blood samples were obtained from healthy volunteers, and buffy coats were obtained from the National Blood Transfusion Service, in accordance with the approval of the relevant Ethical Review Boards. PBMCs were isolated using endotoxin-free Histopaque 1.077 (Sigma-Aldrich) gradient centrifugation. CD14^+^ monocytes were purified using anti-CD14 magnetic beads (Miltenyi Biotec, Bergisch Gladbach, Germany). DCs were generated by culture in DC medium [RPMI 1640, 10% FCS, 1% sodium pyruvate (all from Sigma-Aldrich), containing rhGM-CSF (1000 U/ml) and rhIL-4 (1000 U/ml)] for 5 d. Additional complete medium was added on d 3. The purity and quality of DCs were determined by flow cytometry and morphologic analysis.

### DC-FB coculture

The human dermal FB cell line BJ6 was obtained from Dr. Lloyd Hamilton (University of Nottingham, United Kingdom), whereas primary dermal FBs were obtained from Dr. Anja Saalbach (University of Leipzig, Germany). All cells were tested free of mycoplasma infection before use. For coculture, FBs were seeded in flat-bottomed, 96-well plates and rested overnight. DCs were added to FBs at a 4:1 ratio. Cocultures were incubated for 24 h in a humidified atmosphere of 5% CO_2_ in air at 37°C. Supernatants were collected and stored at −20°C. All experimental conditions were performed in biologic triplicates and on multiple donors. In some experiments, the COX2 inhibitor indomethacin (2 µM) was added to FB before coculture to determine the contribution of PGE_2_ synthesis in the induction of IL-23. For assessment of cell-cell interaction, a Transwell-permeable support system (Corning Life Sciences, Tewksbury MA, USA) was used with FB in the lower and DC in the upper chamber, separated by 3 μm pores. Primary FBs were used up until and including the 4th passage, after which they were discarded.

### Irradiation of cells

Cells were irradiated in tissue-culture plates immediately before DC activation with LPS (500 ng/ml) and IFN-γ (1000 U/ml). For experiments with indomethacin, DCs were activated 3 h before irradiation and adding to FBs to minimize the effect of COX2 inhibitor on DC maturation. Cells were irradiated (0–6 Gy of 195 kVp X-rays, 0.87 Gy/min, 0.5 mm copper filter, 48.4 cm focus to skin distance) using a cabinet irradiation system (Gulmay; Xstrahl, Surrey, United Kingdom). Cell morphology was monitored by phase-contrast microscopy (×40) following IR and again after a further 24 h of culture. Cell viability, apoptosis, and necrosis were determined 24 h after radiation by blue exclusion and annexin V/PI FACS using DMSO as a positive control (not shown). Flow cytometry was performed using an FC500 flow cytometer (Beckmann Coulter, Brea, CA, USA) and analyzed with FlowJo software (Tree Star, Ashland, OR, USA).

### Measurement of secreted cytokine

The secretion of IL-23p40/p19 or IL-12p70 was determined by commercial human IL-23 Ready-SET-Go! ELISA (eBioscience) and human IL-12p70 ELISA kits (BD Biosciences). Assays did not significantly react with other proteins, and the sensitivities were 15 and 7.8 pg/ml, respectively. IL-6 was measured by ELISA (ImmunoTools) with a sensitivity of 9 pg/ml. IL-1β, TNF-α, IL-17, IL-27, and PGE_2_ were measured with DuoSet assays (R&D Systems Europe), and assay sensitivity was 3.9, 15.6, 7.8, 156, and 30.9 pg/ml, respectively. Absorbance was measured at 450 nM using a spectrophotometer.

### Generation of Th17 responses

DC-FB cocultures were treated as described previously for 12 h and then washed with fresh medium to limit the impact of LPS/IFN-γ on T cells. We previously established that IL-23 is not produced by DCs until >12 h after TLR stimulation [35]. Human naïve CD4^+^ T cells were obtained from fractionated fresh whole blood, and naïve CD4^+^ cells were obtained via a 2-step isolation procedure using the Naïve CD4^+^ T cell isolation kit (Miltenyi Biotec). Isolated T cells (CD4^+^CD45RA^+^, purity >95%) were cultured in 48-well plates at 2 × 10^5^ cells/well. Allogeneic T cells were cultured in a 1:1 ratio of fresh medium and DC:FB coculture supernatant for 5 d in the presence of anti-CD3 (OKT3; 1 μg/ml) and anti-CD28 (5 μg/ml) antibodies with IL-2 (50 IU/ml). As controls for Th17 polarization and the importance of IL-23, T cells were treated with rIL-1β, rIL-6, and rIL-23. T Cells were rested for 2 d and restimulated with anti-CD3/anti-CD28. For flow cytometric assessment of Th17, cells were treated with Brefeldin A (BD Biosciences) for 20 h, fixed with 0.5% formaldehyde, permeabilized (Perm Buffer; BioLegend, San Diego, CA, USA), and incubated with mouse anti-human IL-17 FITC (eBioscience). Flow cytometry was performed using Beckman Coulter FC500 flow cytometer and analyzed with FlowJo software (Tree Star). Supernatants from parallel cultures without Brefeldin A were harvested after 48 h for ELISA.

### Measurement of intracellular pATM

For measurement of intracellular pATM, cocultures were separated using 0.3 μm membrane Transwell plates (Corning Life Sciences). FBs were seeded in the lower chamber and allowed to rest overnight before DCs were added to the upper chambers. Cultures were irradiated and activated immediately with LPS/IFN-γ. After 12 h, DCs were fixed in cold 2% formaldehyde/PBS and permeabilized with cold methanol. The binding of primary pATM (Ser1981; eBioscience) antibody was detected with a FITC-conjugated secondary antibody (Dako, Glostrup, Denmark). Cells were acquired using a MACSQuant cytometer (Miltenyi Biotec) and analyzed using FlowJo (Tree Star).

### Quantitation of mRNA level for IL-23A and IL-12B with real-time RT-PCR

Real-time PCR was performed as described previously [33, 34]. In short, cells were plated in DC medium, rested for 1 h, irradiated, and stimulated immediately with LPS/IFN-γ. RNA was isolated (NucleoSpin RNA II kit; Macherey-Nagel, Düren, Germany) and cDNA prepared using the GoScript Reverse Transcription System (Promega, Madison, WI, USA). TaqMan quantitative PCR was carried out for IL-12B and IL-23A with TOP1 as a housekeeping gene (Thermo Fisher Scientific, Waltham, MA, USA; TOP1 Hs00243257_m1, Hs00168405_m1, *IL-12B/IL-12p40* Hs00233688_m1, *IL-23A/IL-23p19* Hs00372324_m1) with MasterMix (Primerdesign, Southampton, United Kingdom) on a Mx3005P (Stratagene, La Jolla, CA, USA) and analyzed with Stratagene software. Quantification was done by the ΔΔC_T_ method, where ΔC_T_ = (gene of interest C_T_) − (TOP1 C_T_); ΔΔC_T_ calculated with mDC at 0 Gy as a reference condition.

### Statistical analysis

Results were statistically analyzed using Student’s *t* test or 2-way ANOVA in GraphPad Prism (GraphPad Software, La Jolla, CA, USA). Significance levels are indicated in the figure legends.

## RESULTS

### Irradiated FBs recover IL-23 secretion from irradiated DCs

**Figure 1.**
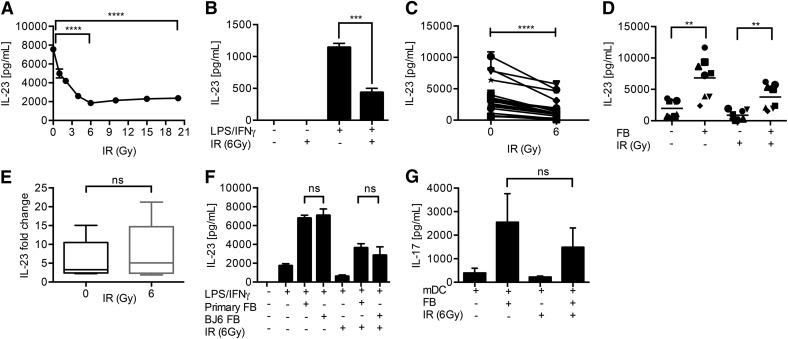
FBs promote the IL-23–Th17 axis during irradiation. IR suppresses IL-23. DCs were irradiated before addition of LPS/IFN-γ and IL-23 release measured by ELISA. (A) IL-23 suppression was dependent on the dose of IR (a representative donor from 3). (B) A representative experiment at 6 Gy. (C) A summary of 18 donors. (D) FBs rescue IL-23 secretion by irradiated DCs. Coculture of irradiated FBs with irradiated DCs up-regulated IL-23 secretion (summary of 8 donors). (E) Fold change of IL-23 secretion by irradiated DC/FB cocultures at 0 and 6 Gy. (F) Primary dermal FBs demonstrate similar IL-23-enhancing activity to the BJ6 cell line (data from a representative donor or 5). (G) FBs permit irradiated DCs to promote Th17 responses. IL-17A secretion by naïve CD4^+^ T cells activated with anti-CD3/anti-CD28 in the presence of supernatants of the indicated DC/FB cultures. T cells were stimulated for 5 d and IL-17A secretion determined after restimulation (collective data from 3 donors). Error bars indicate sd of triplicate experiments. **0.01 > *P* ≥ 0.001, ***0.001 > *P* ≥ 0.0001, ****P < 0.0001; ns, not significant. Results are presented as means ± sd.

The impact of stromal cells on the response of DCs to IR has not been studied previously. Therefore, we tested the hypothesis that DC-FB crosstalk overcomes the immune-inhibitory effect of IR. We recently established that exposure of DCs to IR inhibits TLR4-dependent IL-23 secretion through the activation of ATM kinase [33]. In agreement with this, irradiation of DCs significantly suppressed TLR-dependent IL-23 secretion. Radiation-induced suppression of IL-23 was dose dependent and maximal at 6 Gy (*P* < 0.001; **Fig. 1A**) and occurred in all donors tested (*P* < 0.05; Fig. 1B and C and **Table 1**).

**TABLE 1.** **Effect of IR on cytokine secretion by mDCs**

Cytokine	% Remaining secretion	sd	*P*
IL-23	43	11	<0.0001
IL-12	89	16.3	0.0005
IL-27	100.9	36.6	ns
IL-1β	93.5	34.4	ns
TNF-α	111.8	29.6	ns
IL-6	174.1	114.4	ns
IL-10	95.9	6.2	ns

DCs were irradiated (6 Gy) or nonirradiated before activation with LPS/IFN-γ. Supernatants were collected following 24 h incubation, and secreted IL-23, IL-12, IL-27, IL-1β, TNF-α, IL-6, and IL-10 were measured by ELISA. All experiments were repeated at least 4 times in triplicate, and significance was assessed with 2-way ANOVA.

Coculture of FBs with TLR4-activated DCs (mDCs) markedly increased the secretion of IL-23 (*P* < 0.05; Fig. 1D). Furthermore, we determined the ability of irradiated FBs to sustain IL-23 responses from irradiated DCs. It is noteworthy that irradiated FBs maintained their ability to support increased IL-23 secretion from irradiated DCs (*P* < 0.01) in all donors (Fig. 1D). The levels of IL-23 secreted by irradiated mDCs cocultured with irradiated FBs were greater than or equal to those obtained from nonirradiated mDC controls. It should be noted that whereas IL-23 secretion was increased in the presence of FBs, the levels were nevertheless lower than those obtained from nonirradiated cocultures. However, the fold increase of IL-23 secretion by cocultures was similar regardless of irradiation (Fig. 1E). The ability of FBs to increase IL-23 secretion by irradiated DCs was maintained regardless of whether the BJ6 cell line or primary dermal FBs were used (Fig. 1F). As expected, neither iDCs nor FBs secreted IL-23, irrespective of irradiation status or addition of FBs (data not shown).

In the presence of IL-6 and IL-1β, IL-23 serves a key role in the polarization of human naïve CD4^+^ T cells toward a Th17 phenotype [33, 36]. Therefore, we determined whether the protective effect conferred by FBs on IL-23 production resulted in the induction of Th17 responses. Stimulation of naïve CD4^+^ T cells through CD3/CD28 in the presence of conditioned supernatants from mDC cultures elicited Th17 responses, as shown by secretion of IL-17A (Fig. 1G). Furthermore, the addition of FB to DC enhanced IL-17 secretion by T cells in all donors tested, irrespective of exposure to IR.

### Irradiated DCs maintain their ability to stimulate FBs

To determine the mechanism by which FBs increase IL-23 secretion in irradiated DCs, we tested each step in the DC-FB coculture. Initially, we determined whether the secretion of TNF-α and IL-1β were reduced by IR [2]. In contrast to IL-23, DCs maintained their secretion of TNF-α and IL-1β following exposure to IR (Table 1).

**Figure 2.**
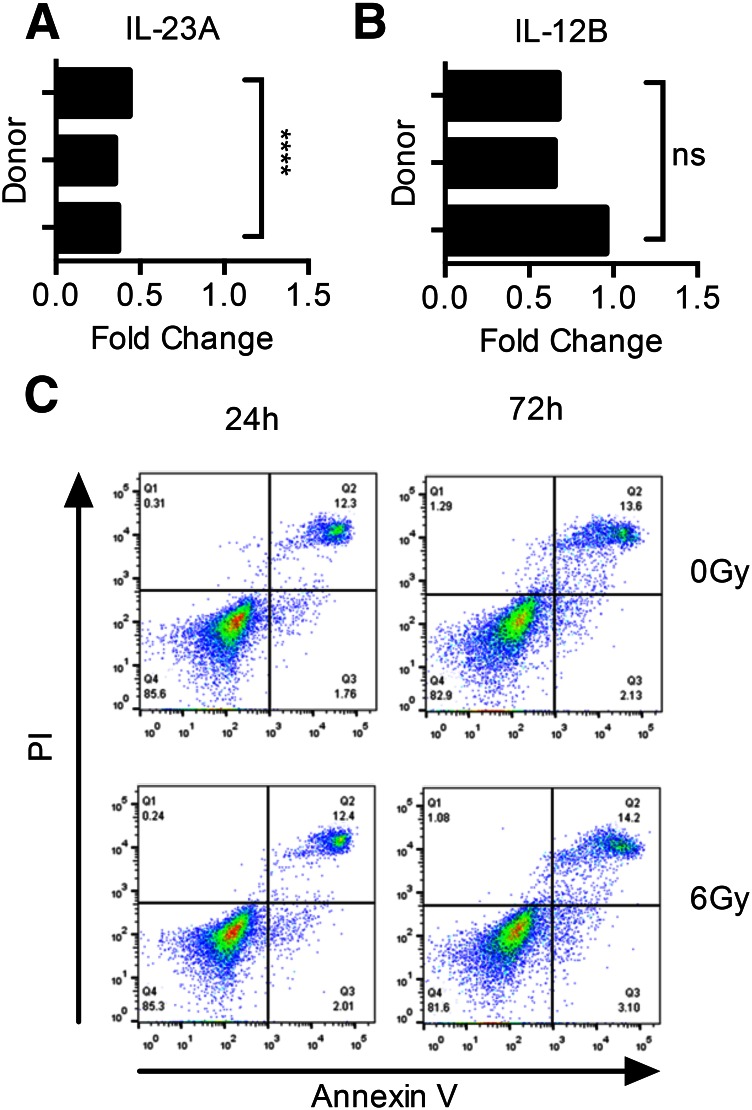
Effect of IR on DCs. Changes in IL-23 secretion were associated with decreased transcription of the (A) *IL-23A* gene and (B) *IL-12B* gene, as shown using quantitative RT-PCR in 3 donors. Statistical significance was measured by the Student's *t* test. (C) Despite exposure to up to 6 Gy IR, there was no decrease in viability of DCs as assayed by annexin V/PI staining with flow cytometry up to 72 h after irradiation. Representative result for 1 of 3 donors tested. ****P < 0.0001. Results are presented as means ± sd.

In view of the differential effect of IR on cytokine expression, we assessed the secretion of other cytokines, including IL-12, IL-27, IL-6, and IL-10 (Table 1). The inhibitory effect of IR was restricted to IL-12 and IL-23, which share a common p40 subunit. IR strongly down-regulated IL-23 and only suppressed IL-12 to a modest extent. Interestingly, another member of the IL-12 family, IL-27, was unaffected. Production of the proinflammatory cytokine IL-6, as well as anti-inflammatory IL-10, was unaffected by IR. Next, we determined whether IR affected transcription of the IL-23-specific *IL-23A* gene or the common *IL-12B* gene that encodes the p40 chain. Irradiation of DCs (6 Gy) significantly suppressed transcription of *IL-23A* compared with nonirradiated DCs (*P* < 0.0001). Although the effect of IR on *IL-12B* was also inhibitory, it was nevertheless less marked than on *IL-23A* (**Fig. 2A** and **B**).

In contrast to many other cell types (e.g., monocytes), DCs remain viable when irradiated [37]. This is because DCs (but not classic monocytes) have constitutively active DNA repair systems that repair double-strand DNA breaks [38]. Therefore, we confirmed that the suppression of IL-23 that we observed in irradiated DCs was not a result of apoptosis or necrosis. IR did not impact the viability of DCs. Staining with annexin V/PI showed the viability of DCs to be to be unaffected by IR (Fig. 2C).

### FBs are functionally unimpaired by IR

**Figure 3.**
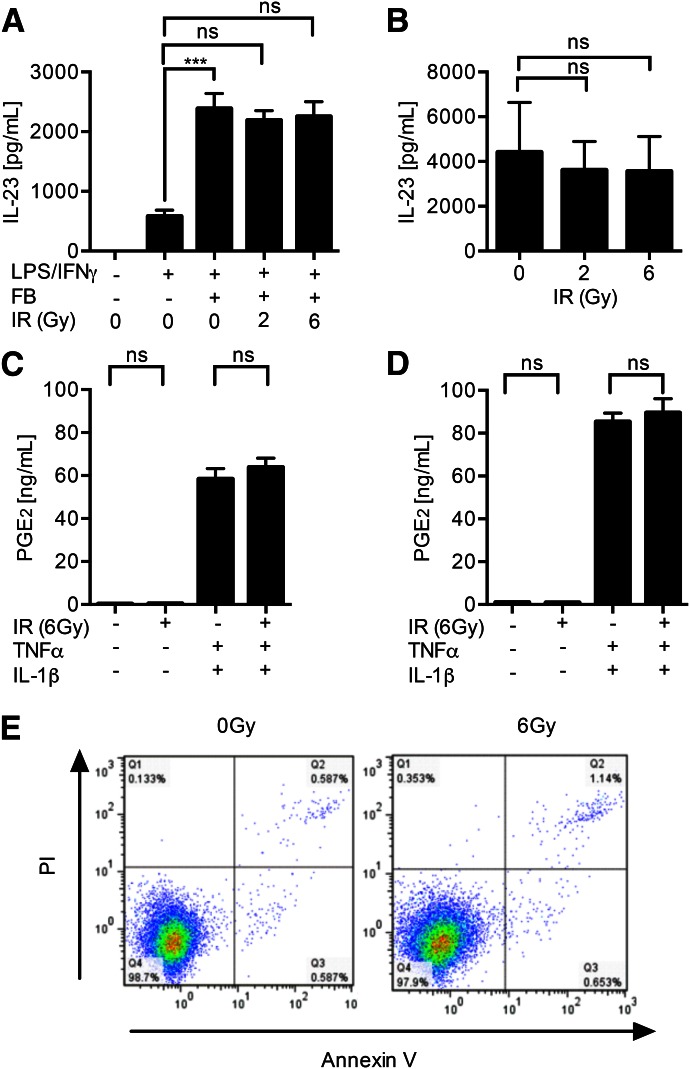
FBs are functionally resistant to IR. Irradiation of FBs did not impair their ability to up-regulate IL-23 secretion from nonirradiated DC. (A) A representative donor. (B) Summary of 3 donors. (C) PGE_2_ secretion (determined by ELISA) by FBs stimulated with rTNF-α and rIL-1β (1 ng/ml) is unaffected by IR; BJ6 cell line. (D) Representative results for 1 of 2 primary FB donors. (E) IR (6 Gy) does not affect FB viability up to 24 h after irradiation, as assessed by annexin V/PI staining; representative data from 3 experiments. ***0.001 > *P* ≥ 0.0001. Results are presented as means ± sd.

As irradiated DCs retained their ability to secrete TNF-α/IL-1β, we next investigated the effect of IR on the response of FBs to these cytokines. Coculture of irradiated FBs with nonirradiated DCs did not impair their capacity to promote IL-23 release from mDCs (**Fig. 3A** and **B**). Furthermore, irradiated FBs retained their ability to respond to signals received from DCs and secrete PGE_2_, as stimulation of irradiated BJ6 and primary FBs with exogenous TNF-α/IL-1β elicited PGE_2_ secretion, which was unaffected by radiation (Fig. 3C and D). As expected, exposure of resting FBs to radiation did not elicit cytokine release. In the absence of DCs, FBs did not secrete IL-6, IL-12, IL-10, IL-17, IL-23, or IL-27 in response to activation with TNF-α, IL-1β, or LPS/IFN-γ, irrespective of exposure to IR (data not shown). Irradiation had no impact on FB viability, as assessed by dye exclusion (data not shown) and annexin V/PI staining (Fig. 3E). It is important to stress that we examined the effects of IR on FBs within the first 24 h of exposure, after which time, activated DCs would be expected to migrate to regional LNs [39].

### FBs support function of irradiated DCs through COX2-dependent PGE_2_ release

**Figure 4.**
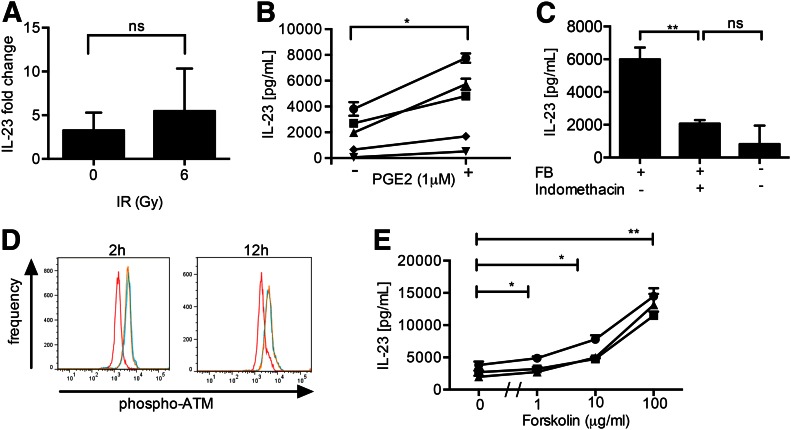
Mechanisms of IL-23 recovery from irradiated DCs by FBs. FB-dependent IL-23 secretion from irradiated DCs is mediated by soluble factors and was independent of cell-cell contact. (A) Fold increase in IL-23 secretion by DCs separated from FBs with Transwell (0.3 μm) treated with IR (summary of 3 donors). (B) Addition of PGE_2_ to irradiated DCs up-regulates IL-23 secretion (summary of 5 donors). (C) Up-regulation of IL-23 secretion from irradiated DCs by FBs is COX2 dependent. FBs were treated with indomethacin for 24 h before (and throughout) coculture with irradiated DCs (data from 3 experiments). (D) FBs did not affect pATM in irradiated DCs. Irradiated mDCs cultured with FBs (blue lines) or without FB (orange lines) show similar levels of pATM. In both of these settings, IR increased ATM activation when compared with nonirradiated mDCs (red lines), as demonstrated by intracellular staining for flow cytometry at 2 and 12 h after IR (6 Gy, representative donor of 3). (E) Stimulation of TLR4-activated DCs with the cAMP agonist forskolin immediately following exposure to IR increased IL-23 secretion (data from 3 representative donors.) *0.05 > *P* ≥ 0.01, **0.01 > *P* ≥ 0.001. Results are presented as means ± sd.

We previously established the importance of FBs for IL-23 secretion by DCs and demonstrated that this was mediated through COX2-dependent PGE_2_ [3]. To assess whether this mechanism was involved in irradiated cells, we first demonstrated the involvement of FB-derived soluble factors. FBs were separated from DCs by a 0.3-µm porous membranes in Transwell plates. Despite the lack of cell–cell contact, FBs retained their capacity to support IL-23 secretion from irradiated DCs (**Fig. 4A**). Therefore, we evaluated the involvement of PGE_2_ in up-regulation of IL-23 secretion by irradiated DCs. The addition of PGE_2_ to DCs immediately after irradiation and activation resulted in a significant increase of IL-23 secretion in all donors tested (Fig. 4B). As PGE_2_ secretion by FB is usually COX2 dependent [2, 37], we determined the importance of COX2 activation for FB-dependent recovery of IL-23 secretion using the COX2 inhibitor, indomethacin. IL-23 production from irradiated cocultures was reduced significantly by indomethacin (Fig. 4C). In the presence of indomethacin, IL-23 secretion from DC–FB cocultures was reduced to levels similar to those achieved in the absence of FB.

### FBs promote irradiated DC IL-23 responses through the cAMP pathway

Irradiation of human DC monocultures selectively inhibits IL-23 by pATM kinase [33]. However, PGE_2_ also up-regulates IL-23 through activation of the cAMP–PKA signaling pathway [40, 41]. Interestingly, in lung cancer, cAMP signaling inhibits IR-induced pATM [42]. Therefore, we sought to dissect the molecular mechanism responsible for IL-23 secretion in irradiated DC-FB cocultures.

Initially, we assessed ATM activation [3] in irradiated DC monocultures or DC-FB cocultures. DC-FB cocultures were separated by 0.3 µm porous membrane to ensure that pATM levels were measured only in DCs. As expected, irradiation of mDCs resulted in pATM after 2 h, and this persisted for at least 12 h, as demonstrated by intracellular staining with flow cytometry (Fig. 4D). However, coculture with FBs did not suppress the levels of ATMs expressed by irradiated DCs, suggesting that the support of DC function by FBs occurred independently of ATM kinase. Lastly, we examined the involvement of the cAMP pathway in regulation of IL-23 from irradiated DCs. The addition of the cAMP active analog forskolin to irradiated monocultures of mDCs resulted in a dose-dependent increase in IL-23 (*P* < 0.01; Fig. 4E), implicating a role for the cAMP signal transduction pathway.

## DISCUSSION

Studies on the impact of the stromal microenvironment on immunity are important as immune cells are in constant crosstalk with their stroma during each and every stage of the immune response. Stromal cells have the ability to affect a wide range of immune functions, including DC maturation, their migration to LNs, and subsequent polarization of T cell responses [1, 2, 12, 43]. The present study investigated the ability of stroma to modulate the outcome of therapeutic interventions directed toward the immune system. Our previous work established that IR inhibits the cytokine response of DCs and in particular, IL-23 [33]. On the other hand, stromal FBs support IL-23 production by activated DCs [2]. Therefore, we addressed the hypothesis that FBs, cells that are known to be relatively radio resistant [44], continue to support the function of DCs in the presence of IR.

The doses of IR used in the current study are similar to those used during routine RT of common malignancies [26, 29]. At these doses, IR damages transformed cells and initiated their demise by generating free radicals that induce stress responses and consequently, cell death if the damage is not repaired [29, 38, 45, 46]. As a result of their high proliferative rates and impaired DNA repair mechanisms, many tumor types are selectively sensitive to radiation-induced DNA damage [30, 47]. However, whereas tumor cells are the main target for RT, immune and stromal cells residing in the tumor microenvironment are also exposed to and affected by IR [48]. In this regard, previous work using monocultures has shown that IR suppresses IL-12 secretion by MoDCs between 2 and 20 Gy [31], whereas another report showed increased IL-12 secretion by murine DCs at 0.05 Gy, and this effect was reversed to the level of nonirradiated DCs at 1 Gy [49]. The disparity between these findings may be caused by differences in the behavior of human and murine DCs; however, they may also reflect different mechanisms of action for IR at extremely low doses (0.05–1 Gy) compared with higher doses used for RT (2–6 Gy) [26, 50].

We found that the inhibitory effect of IR was restricted to IL-12 family members sharing the common p40 subunit. However, the effect was considerably more pronounced for IL-23 than IL-12p70. Interestingly, the maximal effect of IR was exerted at 6 Gy with a plateau of effect at higher doses. This may be a result of ATM reaching its maximum activation state at this dose. On the other hand, the effect of IR did not extend to the other IL-12 family member, IL-27, comprising the IL-27p28 subunit and EBV-induced gene 3, which are related to p35 and p40, respectively [51]. Those findings demonstrate the highly selective effect of IR on DC functions with a prevalence to inhibit Th17 responses. The lack of effect of IR on TNF-α and IL-1β had important consequences for our multicellular model, as it allowed activated DCs to sustain their interactions with FBs and generate the PGE_2_ feedback loop [3]. Irradiated FBs also continued to support the production of IL-23 by DCs, irrespective of the dose of IR to which FBs were exposed. This was observed not only with an FB cell line but also with primary dermal FBs, thus supporting the potential physiologic importance of this observation.

FBs (resting, TLR activated, or irradiated) were not responsible for the secretion of any of the cytokines affected by IR. Previous reports describe FB expression of the IL23p19 subunit upon stimulation with IL-1β [52], but to the best of our knowledge, there are no reports of IL-23 heterodimer secretion [53]. It is important to recognize the difference between biologically active IL-23 heterodimer secreted by APC and IL-23p19 monomer, and in the current study, we measured heterodimeric IL-23 secretion [53].

It is known that FBs can enter a senescent phase when exposed to IR yet remain viable [18, 44, 54]. This represents 1 of the important mechanisms of tumor development, as in this state, senescent FBs alter their phenotype and promote tumor growth and invasion and render adjacent tumor cells increasingly radio resistant [44]. Therefore, we assessed the effect of IR on FBs in functional assays. PGE_2_ stimulates IL-23 secretion in human and murine DCs [2, 41, 55, 56]. Previous reports have published conflicting data regarding regulation of PGE_2_ secretion by gingival FBs irradiated with a low-level diode laser [57, 58]. In our model, irradiation of dermal FB did not alter PGE_2_ secretion in response to exogenous TNF-α and IL-1β, and this likely accounts for their continued capacity to augment IL-23 release by nonirradiated DCs. Therefore, irradiated FBs were unimpaired in their ability to secrete soluble immune modulators and maintained their capacity to respond to environmental stimuli.

According to recent reports, PGE_2_ stimulates IL-23 secretion from DCs through activation of the cAMP/PKA pathway [40, 41], and we previously described that IR down-regulates IL-23 through pATM kinase [33]. Interestingly, cAMP signaling inhibits radiation-induced pATM in lung cancer [42]. Addition of forskolin increased IL-23 secretion by irradiated DCs to levels commensurate with those from irradiated DC-FB cocultures, suggesting a role for cAMP. However, in contrast to Cho and colleagues [42], coculture of irradiated DCs with FBs did not affect pATM levels, despite up-regulated IL-23. This disparity may be a result of differences in timing of cAMP and ATM activation. Cho and coworkers [42] activated cAMP before irradiation of cells, thus preventing pATM by activated protein phosphatase 2A. In our study, DCs were irradiated before activation and subsequent stimulation from FBs. As ATM is phosphorylated in DCs within 15 min of IR [33], signals from FBs are received by DCs too late to inhibit ATM activation. Therefore, it is even more notable that up-regulation of IL-23 secretion by FBs occurred despite ATM activation.

The impact of FBs on the ability of irradiated DCs to promote Th17 responses was examined. Previously, we [2, 33] and others [56] established the role of IL-23 in the generation of Th17. In agreement with this, IL-17 secretion from T cells conditioned with supernatants from DC-FB cocultures was enhanced compared with supernatants from DC monoculture. PGE_2_ was shown to promote Th17 directly and in conjunction with IL-23 [59, 60]. In our model, the concentration of PGE_2_ secreted by FBs was unaffected by IR. On the other hand, changes in IL-17 reflected differences in IL-23 levels, demonstrating the biologic importance of FB-dependent IL-23 secretion in regulation of adaptive immunity.

**Figure 5.**
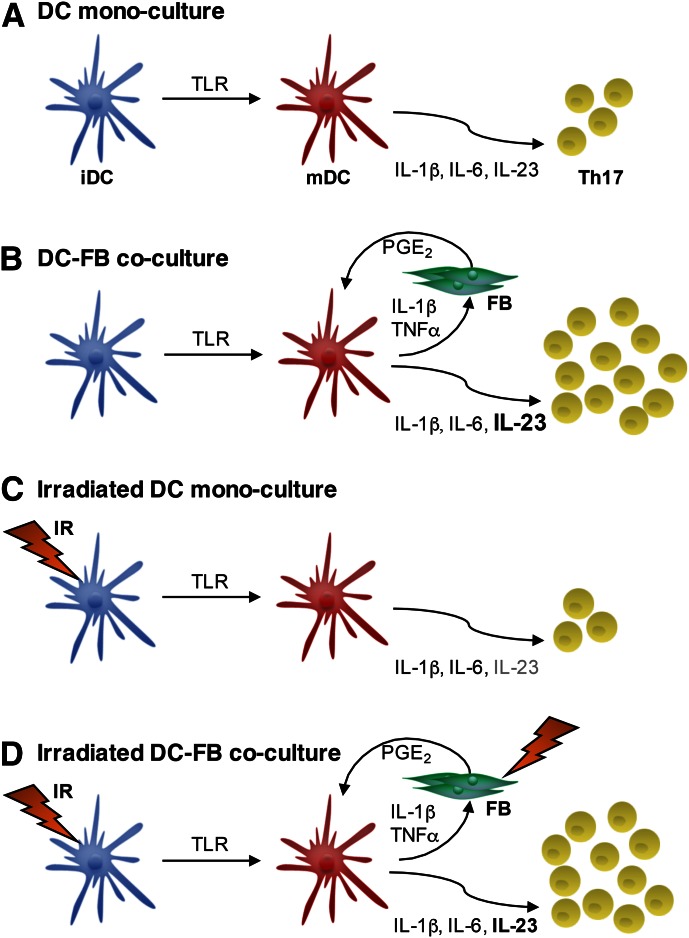
Model of DC-FB crosstalk governing IL-23-dependent Th17 responses after irradiation. (A) TLR activation of DCs in monoculture elicits secretion of IL-1β, -6, and -23, which prime Th17 responses. (B) The addition of FB to DC provides an important feedback loop that serves to enhance IL-23 secretion and thus, augments Th17 responses. (C) Irradiation of DC monocultures selectively inhibits IL-23 secretion. (D) However, the presence of FBs ensures that irradiated DCs continue to secrete sufficient IL-23 to generate Th17 responses. Importantly, the irradiation of FBs does not hinder their reinforcement of IL-23 responses.

In summary, we show that although IR inhibits IL-23 secretion from DC monocultures, the inclusion of FBs provides a positive-feedback loop that serves to maintain IL-23 secretion by DCs. We propose that this translates to enhanced Th17 responses in a post-RT environment (**Fig. 5**), and studies to investigate this are underway. As IL-17 is a critical factor driving postradiation fibrosis [24], this work identifies a potential mechanism for the pathologic consequences of radiation and chemotherapy [25, 61]. Importantly, it highlights the need for multicellular models of the immune microenvironment. Insight into the complex interactions between the immune system and stroma is necessary to our understanding of pathology and for development of novel therapeutic interventions.

## AUTHORSHIP

A.M. undertook or supervised all experimental work, interpreted the data, and wrote the manuscript. Q.W., S.S., R.V.S., and H.A.F. undertook specific components of experimental work. A.S. generated and characterized primary dermal FBs and wrote the manuscript. I.S., J.M.R., J.G., and M.J.G. undertook statistical analysis and wrote the manuscript. M.J.G. provided advice on radiation responses. P.M.P. conceived of the original project idea and supervised the research. A.M.J. conceived of the original project idea, supervised the research, and wrote the manuscript.

## Abbreviations

ΔΔC_T_: comparative threshold cycle
ATM: ataxia-telangiectasia mutated kinase
COX2: cyclooxygenase 2
DC: dendritic cell
FB: fibroblast
iDC: immature dendritic cell
IR: ionizing radiation
LN: lymph node
mDC: mature dendritic cell
MoDC: monocyte-derived dendritic cell
p: phosphorylated
PI: propidium iodide
PKA: protein kinase A, rh recombinant human
RT: radiotherapy
TOP1: topoisomerase 1
